# Micro-Nano Plastic in the Aquatic Environment: Methodological Problems and Challenges

**DOI:** 10.3390/ani12030297

**Published:** 2022-01-25

**Authors:** Saif Uddin, Scott W. Fowler, Nazima Habibi, Montaha Behbehani

**Affiliations:** 1 Environment and Life Sciences Research Center, Kuwait Institute for Scientific Research, Safat 13109, Kuwait; nhabibi@kisr.edu.kw (N.H.); mbahbaha@kisr.edu.kw (M.B.); 2 School of Maine and Atmospheric Sciences, Stony Brook University, Stony Brook, NY 11794-5000, USA; fowlerscottw@yahoo.com; 3 Institute Bobby, 8 Allée des Orangers, 06320 Cap d’Ail, France

**Keywords:** microplastic, nanoplastic, vector for contamination, environmental concentrations

## Abstract

**Simple Summary:**

The topic of plastic wastes, microplastic (MP) and nanoplastic (NP) particles in the aquatic environment has been the focus of much scientific effort over the last decade and has gained immense public attention through the media. While numerous scientific reports underscore the ubiquitous presence of MPs and NPs in aquatic environments, particularly the oceans, there are many unresolved issues involved in their sampling, identification and characterization. This paper addresses some of the main problems and suggests what needs to be undertaken to overcome these issues. An overriding problem is the lack of harmonization of the protocols used for sampling MPs at sea and identifying them in the laboratory. There are technological challenges in polymeric characterization of NPs in environmental samples. Researchers use a wide variety of net types and net mesh sizes to capture and separate MP floating in the sea which makes comparing MP concentrations from different teams and areas extremely difficult and calls for establishing inter-comparison exercises among the various research teams. Furthermore, the issue of whether chemicals in MPs and NPs leach following ingestion by biota or whether they transport and release contaminants adsorbed on MP/NP surfaces is still unresolved. In essence, these and other issues have to be addressed and resolved before society has an accurate picture of their importance as an aquatic pollutant. There are no datasets on the environmental concentration of NPs, hence their effect on biota is solely relied on laboratory experiments using extremely high concentrations. The legitimacy of these effects and interactions in the biotic system is something that warrants discussion.

**Abstract:**

Microplastic research has become a buzz word. It is seen as one of the most pressing issues of Anthropocene contamination. There is certainly no doubt about the ubiquitous presence of microplastic (MP) in almost all environmental matrices. However, the validity of considering them as a vector for contaminants needs some reconsideration, there are other more potent pathways. Their effect on marine biota also calls for some realistic experiments with environmental concentrations of MP and nanoplastic (NP). It has been observed that in most published literature, polymer characterization is performed. Is it necessary to do, or will merely finding and confirming the particle as plastic suffice for environmental research? Harmonization of protocols is necessary, and there is likely a need for some inter-laboratory comparison exercises in order to produce comparable data and reliable assessments across regions. Samples collected from the same area using different techniques show an order of magnitude difference in MP concentration. The issue of nanoplastic is more contentious; are we technologically ready to identify NP in environmental samples?

## 1. Introduction

Plastics are the most diverse material humans have produced and in enormous quantities; during 2020, amongst COVID-19 lockdowns and global slowdown, 367 million tons were produced [1]. The omnipresence and persistent character of microplastic (MP) [2] have attracted significant scientific interest globally. A bibliographic search for microplastic and nanoplastic (NP) using SCOPUS on 30 May 2021 returned 6551 articles, including 5163 research papers, 124 book chapters, 274 conference proceedings, and 697 reviews articles, similar information was reported in much more detail by Palmas et al. 2021 [3]. The majority of these communications were on MP in the marine environment, and a few were on MP in freshwater [4]. Moreover, there were fewer than three dozen on MP in the aerosol. With so much focus, microplastic (MP) has become a major research topic of environmental and ecological concern in aquatic environments [5]. Microplastics have been found in all environmental matrices including water, suspended particulates in the water column [6,7,8,9,10,11,12,13,14,15,16], sediments [6,17,18,19,20,21,22,23,24,25,26,27,28,29,30,31,32,33,34], wastewater [35,36,37,38,39,40,41,42,43,44,45,46,47,48,49,50,51,52,53,54], biota at different trophic levels [19,21,55,56,57,58,59,60,61,62,63,64], aerosols [65,66,67,68,69,70,71,72,73,74,75,76,77,78,79] and even in snow [80]. However, information is very limited for large surface waters and almost absent for smaller surface water bodies [4].

The origin of MP in the aquatic environment is often linked to the colossal quantity of plastic entering mainly from poorly managed plastic waste disposal [32,41,47,53,81,82]. The chronic exposure of MP in the aquatic environment poses various issues such as inducing inflammation, malnutrition, reproductive behavior and capacities [57,83,84,85,86,87,88,89], and toxicity caused by the polymer used for manufacturing plastic products [60,90,91,92,93]. The potential of MPs to adsorb hydrophobic contaminants due to their extremely large surface-to-volume ratio and long residence time in the water column [94,95] has attracted much attention in the scientific community [7,16,32,47,53,70,71,87,96,97,98,99,100,101,102,103,104,105,106,107,108,109,110,111]. The primary ecological concern from MP emanates from the fact that many aquatic organisms across the trophic food chain misidentify them as prey. Continued ingestion may lead to malnutrition, as well as the transfer and bioaccumulation of toxic chemicals [112] by MP as a carrier, and via leaching of chemicals from the MP that might affect their physiological functioning.

The MP in the environment occurs both as ‘primary’ or ‘secondary’ particles. The primary MPs are manufactured for incorporation into other products, while those that break down from larger plastic debris to a size <5 mm are considered secondary MPs. Studies have investigated the pathways of MPs reaching the aquatic environments; a recent study has highlighted the role of precipitation and aeolian transport in transporting both the primary and secondary MPs [38], in addition to the wastewater treatment streams [38,47,50,53,113]. The MPs exist in various shapes as fibers, films, fragments and spheres, in sizes varying from 1–5000 µm. The lower size range of 1 µm is the smallest particle where the polymer can be characterized using micro-Raman spectroscopy [114,115,116]. In comparison, micro-FTIR can be used to identify polymers in particles >10 µm in size [117,118,119,120].

There is significant chemical and physical diversity among the MPs present in the environment. Rochman et al. [112] suggested MPs be considered as a suite of contaminants rather than as a single contaminant [121]. Another new term that has come up in a few publications is nanoplastic (NP), a widely acceptable size range for NP is between 1 nm and 100 nm [122]. Still, the amount of nanoplastic in the environment is not known, mainly due to technological limitations; however, significant efforts have been made by undertaking laboratory experiments to assess the toxicity of NP to biota [123,124,125,126,127,128,129,130,131,132,133,134,135,136,137,138,139,140,141,142,143,144,145,146,147,148,149,150,151,152,153]. Most of these experiments are conducted at much higher concentrations (non-environmental). Sometimes the information is misrepresented as well, a recent review in toxicological research of nanoplastics in the environment [154] reported “nanoplastics can act as carriers of chemical and biological contaminants”; however all of the three references used to support the statement were on microplastics [155,156,157]. This is not the only case, there are hundreds of such misreportings in MP-NP literature where authors have conveniently and interchangeably used debris, MP and NP, although they are very different things and require an entirely different approach to assessment. There are enormous technological challenges associated with NP sampling, sample preparation, identification and characterization. Although their presence in environment is quite eminent due to their use in cosmetics and personal care products, some workers have suggested that decomposition of MP will continue to form NP [158] as well.

## 2. Current Status of Microplastic in Aquatic Environment

The bulk of the >6500 scientific papers published on micro- and nano-plastics usually report the occurrence of MPs in specific aquatic systems, their uptake in the marine food chain, and inventories in sediment and much fewer on NP. These studies have helped in defining the scale and severity of the problem. Only a few papers have raised the issue of the need to have standardized protocols for sample collection, preparation, identification, and reporting units. Several attempts have been made towards harmonization of methodologies [9,25,33,88,111,159,160,161,162,163,164], and we would like to summarize some of these issues according to different matrices in which MP and NP are studied, in addition to a general discussion common across the matrices.

### 2.1. Seawater

Some of the frequent issues observed across many of the published MP research papers start from the collection of samples. The volume reduction using Manta, neuston, or plankton nets is the most commonly used method for MP sampling in seawater [9,11,12,100,103,111,165]. These samplers have their own design biases; the Manta and neuston nets have rectangular openings. Manta nets are equipped with stabilizers that keep them at the surface, partly submerged, and samples are collected from the top 15–25 cm [111]. Neuston nets collect a slightly deeper sample from 40–50 cm depending on the opening size. The plankton nets are circular, usually 30–60 cm in diameter, and collect samples from the top 30–50 cm. Does this mean heavier MPs are quite likely to be missed because only a thin veneer of surface water is sampled using these techniques? Is the higher frequency of PE and PP observed in seawater samples a result of this bias?

Another factor that can lead to bias in MP enumeration and size is the mesh size of the sampling gear. Most gear comes in different mesh sizes varying between 20 and 3000 µm. Despite the agreement on the lower cut-off size of 1 µm for MPs [33,166,167], guidelines such as the marine strategy framework directive (MSFD) recommend using a 333 µm plankton net for sampling and a mouth aperture of 60 cm [11]. Will this leave considerable room to miss sampling the smaller MPs that are known to represent 35–90% of all MPs present in the marine environment [33,111,168]? It is evident from published literature, the most commonly used mesh size for MP sampling in the aquatic environment varies between 150–333 µm, leaving behind a significant portion of fine MPs [169]. So, can we compare these MP concentrations across the published literature without correcting the bias due to mesh size, and is this correction possible?

The third significant bias introduced while reporting the concentration of MP comes from the fact that not many sampling gears are equipped with flow meters. The volume computation is performed using sampling duration and towing speed or sometimes from two GPS locations to compute the distance covered. However, none of these account for current velocity and direction, and whether the sampling gear was submerged or not, and therefore can introduce significant errors in volumetric estimates. Several studies have reported MP m^−2^, while others have been reporting as MP m^-3^.

Another bias that is introduced is when a sample is retrieved. Ideally, the entire sampling gear should be washed from outside using freshwater so all the material is flushed into the cod-end, which should be transferred into a clean glass jar. Some workers have not been doing such cleaning onboard, which can lead to carryover from one site to another if the same gear is used for collection without proper cleanup.

The segregation of microplastic from the seawater sample is a vital preparation step. Most of the researchers have used filtration of the sample on filter paper. The choice of the filter paper is quite critical; the most commonly used is glass fiber filter paper, but some studies have reported using nylon and PFTE filters as well. Whether these different filtration methods can introduce contamination remains a contentious question that needs some debate and consideration from the experts. Most studies use a 0.45 or 1 µm filter, even when the samples were collected using 333 µm net gear. A possible explanation for using the fine filter may be capturing smaller MPs that might be adhered to the phyto- and zooplankton or even ingested by smaller micro-organisms or in fecal pellets. Density separation is a critical step for MP segregation from other co-deposited particles. The effectiveness of the density separation depends on the type of solution used; NaCl is most commonly used. With a density of 1.2 g cm^−3^ it can float most but not all the polymers, whereas KI, NaI can result in better segregation, while ZnCl_2_ can float all the commonly found polymers [16,53].

Sample preparation is a crucial step and some workers choose to filter all the solutions and liquids through a 1 µm pore size filter, while most workers use non-filtered solutions for density separation, such a process can introduce MP from secondary sources, especially when common salt and tap water is used [170,171,172,173,174,175,176,177,178,179,180,181].

Microplastic associated with organic matrices, fecal matter, bio-fouled or covered with biofilms is not uncommon. The most commonly applied treatment for organic matter removal is digestion in 30% H_2_O_2_; many studies report heating the sample to 60–70 °C. All of this poses the question of will such a treatment obliterate the physical and chemical characteristics of MP? There are informed advisories on using 15% H_2_O_2_, however, the question is will this treatment lead to a false positive or will it make polymeric characterization difficult?

Post digestion, the material is filtered again, sometimes using nylon and polyether ether ketone (PEEK) filters. Others use anodisc/nitrate/silicon membrane filters, or stainless steel, silver, and gold-coated membrane filters since these can be directly used for micro-Raman or micro FTIR analyses. Several workers have noted using Nile Red for staining the MPs for identification under UV-Microscope. Unfortunately, if it was composed of nylon and PEEK, the filter will be stained as well. Some of the workers have been using a scanning electron microscope (SEM) for MP and smaller MP identification which is an invasive technique, and the sample has to be coated with metal. If this is the case, no polymeric characterization is possible on this type of sample.

### 2.2. Freshwater

The sampling procedures and processing steps are not any different between seawater and freshwater. Microplastic in the freshwater system was first reported from Lake Geneva [182]. The MP in freshwater systems has covered both lotic and lentic waters [183]. MPs have been reported from surface water from Oceania, Africa. North America, South America, Europe and Asia [184]. Another discrepancy that is quite obvious in the case of freshwater is units of measurements, some authors have used MP kg^−1^, MP L^−1^, MP m^−3^, mg km^−2^, MP km^−2^, and g m^−3^.

### 2.3. Marine Sediments

The beach sediments are the most studied matrix for MP abundance. However, authors have been using different sections of the beach, some have covered the entire beach transects (perpendicular to the shoreline) while others have sampled specific littoral zones. However, the lack of uniformity between studies explains why the MP distribution on beaches is still poorly understood.

It is prudent to mention that most studies report single sampling campaigns from the intertidal zone, however, this is a dynamic zone that represents significant seasonal variation between the high and low water lines and undergoes continuous changes. Some protocols have indicated marking out a 100 m line, parallel to the seawater’s edge [25] and therefore samples are collected on either side of this demarcation.

Another matrix commonly studied is the bottom sediment. Authors have used various types of samplers (van Veen grab, barrel type sediment gravity corer, a box corer, or scooping by divers). The choice of sampler should depend on the objective of the study, type of sediments in the study area, bathymetry and hydrodynamic conditions. Some of the commonly used methods are discussed here. A sediment core using a box corer was collected in the Santa Barbara Basin, southern California at ~580 m depth [185]. In other studies from a canal in Tokyo Bay and Sakurada-bori Moat in Japan, the Gulf of Thailand, Straits of Johor, Malaysia, the Durban Bay, South Africa, sediment cores were collected using an 11 cm diameter gravity corer [186]. In another study, a box corer was used to collect samples from southern China [187] and the Irish continental shelf [30], while a piston corer was used to obtain samples from the Donghu Lake, Wuhan City, China [188] and 6 cm diameter gravity corer for collection of samples from the Andong Salt Marsh in Hongzhou Bay, China [189]. Some very deep samples from the Challenger Deep [31], 29 sediment cores from the Levantine, Catalan, Alboran and Cantabrian basins within the Mediterranean Sea [190], and from the Arctic [191] were collected using a multicorer. Sediment core samples were also collected in shallow marine sediments off Samos Island, Greece using a stainless steel hand corer [192].

Similarly, authors have used several different approaches to establish chronology in sediment cores from x-radiograph and color imaging, polychlorinated biphenyls (PCBs) peak concentrations, and ^14^C, ^137^Cs, and ^210^Pb dating. Several workers have collected the cores but have not attempted establishing chronology in sediment cores. Reviewing these studies raises a few concerns: when a sediment core is collected, why was it not dated? Sometimes a 2 cm or thicker slice of core was dated, was this because there was insufficient material for gamma spectrometry? If the authors use a wider diameter core or box corer, they can get a better temporal resolution. Use of ^14^C for recent sediment dating, when MPs are known to be in the less than 100 years horizon, the type of models used for historical reconstruction (constant rate of supply (CRS), constant initial concentration (CIC)), present some issues that suggest advance planning and a multi-disciplinary team are required when such attempts are made.

It is noteworthy that unfortunately not all the studies have employed grain-size characterization (sand, silt, clay), as this can be a reliable indicator of the prevalent hydrodynamic regime at the time of deposition and will have a significant bearing on MP retention. Some bottom sediments have a high organic matter content, and therefore will require a pre-treatment. Several workers have been using 30% H_2_O_2_ and density separating the MPs, while others have suggested the superiority of KOH [193]. Again, depending on the type of solution used, all MPs might not become buoyant, i.e., PC, PU, alkyd, polyester, PET, PE, PVA and PTFE are denser than NaCl which is the most commonly used solution for density separation. Some workers have used KI (1.62 g cm^−3^) which is quite effective with the exception of PVA and PTFE. Very rarely people have used ZnCl_2_ which can float all sorts of plastics but is cost-prohibitive compared to NaCl. Several workers have not used Nile Red, or any other florescence dye which can make it difficult to distinguish between smaller MP fragments and mineral grains, glass and natural fibers.

### 2.4. Biota

There is an exponential increase in the number of studies looking at the presence of microplastics in marine and aquatic biota [17,19,57,59,60,81,83,89,102,194,195,196,197,198,199,200,201,202,203]. Microplastics find their way into biota usually by ingestion (active uptake) [102,204,205] or adhesion, adsorption, or inhalation (passive uptake). The MPs have been studied in fish, invertebrates and plankton, and most of the published data show the presence of fibers and fragments with very few reports of beads [5]. The field studies have indicated a positive correlation between the MP in biota and MP concentration in the surrounding environment [5,206]. However, most of the biota studies target the organism only, and the environmental concentrations are rarely reported. The work on microplastic as a vector for toxins has attracted huge interest, and there is evidence in the published literature both for and against. A few points to consider are while MPs are very complex chemical products, much of the chemical additives are not part of the polymer [207] and can leach into the environment as potentially toxic substances. Considering that secondary microplastics are often a result of physico-chemical degradation of macroplastic and litter; will this lead to leaching of some of the potentially toxic substances? How much will be leached? These are some questions that do not have very clear answers yet. Another point to ponder is that most of the experimental work performed so far on the toxicity of MPs and NPs has used a significantly higher concentration compared to those in the environment.

The diversity in types of marine biota requires several different types of plankton nets, with different mesh sizes, different volumes of water collected, making any comparison between studies unrealistic. Often this type of information is not provided in manuscripts or supplementary data, sometimes the samples have both zooplankton and phytoplankton together and, if they are not fixed in the field, there is a good chance that some of them will prey upon others in the sample. In fish and crustaceans, most of the ingested MPs are egested fairly quickly, i.e., between one to 72 h. However, there are reports of retention of MPs as long as 40 days [208]. A study on the exposure of MPs in gastropods showed clearance between 12 h and seven days [209]. Again, this depends on the size of the polymer, exposure concentration and duration in most cases these were nowhere near to environmental concentration of MPs.

Another conspicuous issue observed is the number of replicates, and a significant variation in the MP concentration between individuals also indicates that retention within the tissues is not very high. Most of the authors studying MP have looked at the digestive system and gut content, where the MP concentrations are high, again suggesting that they are not necessarily retained within the tissues.

The sample preparation for MP assessment in biota is very critical. Usually, for assessing MP ingestion the target tissue will be the gastrointestinal tract, however, some workers have also looked at other organs. The tissue is usually digested using KOH or H_2_O_2_; the use of concentrated solutions can also degrade the polymer. Several workers use Nile Red for staining, while others use microscopic examination without fluorescent dye.

Researchers have started discussing nanoplastics, a topic that raises the question about the technological limitation of polymer characterization of individual NP particles. The issue of polymer misidentification in MPs using microscopy varies from 33 to 70% [5,103,104,210,211,212,213,214]. Considering this fact, reliable identification of NPs from environmental samples is a legitimate concern.

## 3. Current Perspective of Animal Health and Human Food Safety

A large volume of literature has evolved around the microplastic and nanoplastic risk to animals at different trophic levels. Several studies have reported the presence of MPs in the stomach and gut of various species [215,216,217,218]. Some other studies have reported the translocation of MPs and NPs to other tissues outside the digestive system by fragmentation [218], intracellular or paracellular endocytosis [59], and translocation of small-sized particles [219]. The transfer of 80 μm size MPs along gill filament channels of mussel into the mouth and into the digestive gland was demonstrated by Von Moss et al. [220], and further suggested that translocation into cells could occur. Other evidence of MP translocation in mussels came from the presence of 3 and 9.6 µm polystyrene particles in hemolymph and their prevalence inside hemocytes after 48 d [221]. Experimental results have demonstrated the accumulation of 0.25 μm NPs in the intestine of *Pecten maximus* after 6 h of ingestion, and 24 nm NP in the whole body, indicating possible translocation across epithelial membranes [222]; however, the authors were cautious in confirming such claims. With the growing evidence of the MP and NP presence in the digestive system, liver and gills [62,64,197,202,223,224,225,226,227,228,229,230,231,232], it is postulated that their presence may affect the physiological functioning of the organism, with more evidence coming on ubiquitous presence of MPs [233,234,235,236]. However, no conclusive proof exists on the extent to which the MPs–NPs affect different marine organisms, and much of the evidence emanates from laboratory exposure experiments that show impacts on feeding, oxidative stress, inflammations, and compromised reproductive capacities [237]. It is noteworthy to highlight that most of these exposure experiments use virgin microspheres and significantly higher doses compared to environmental concentrations. Hence, just how reliable and relevant these laboratory exposure experiments are has recently been the subject of debate [237].

Several studies have drawn attention towards MP ingestion via seafood and the likely food safety issues involved [57,238,239,240]. There are over 130 articles published on human exposure to microplastics via ingestion, inhalation, and dermal contact. In humans MP exposures are likely to cause toxicity, however, few studies have demonstrated the potentiality of metabolic disturbances, neurotoxicity, and increased cancer risk. In any case, the epidemiological data and evidence are insufficient to quantify the effects of MPs on human health and their pathogenesis [241].

## 4. Some Common Issues That Call for a Discussion

There are many issues related to microplastic research raised in various reviews mainly targeting the problems related to sample collection, sample preparation, reporting units, non-harmonized protocols, and cross-contamination to name a few.

We believe the first and foremost issue that needs discussion is how serious is microplastic as a pollutant and as a vector of pollutant transport in the environment? Are we overzealous in considering MP as one of the more important issues of Anthropocene pollution? Such questions are evident from the over 6000 papers published in mainstream journals during the last decade.

We would like to draw attention to the fact that concern emanates from the fact that MPs are known to be physically and chemically stable, and once created they remain in the ecosystem for a very long time. It is known that over 4000 chemicals are currently used in the plastic industry, and most of these are additives to improve plastic’s applications. They are often not strongly chemically bound and can readily leach into the environment, certainly a matter of great concern. By the time the primary plastic debris breaks down into MP, are these loosely bound chemicals still present? What is the likely concentration of these chemicals on MP compared to that in the original product, and in the surrounding environment? The answers to these questions will give a good perspective on the seriousness and scale of the threat?

We need to be mindful that MP concentrations in most aquatic environments (away from wastewater treatment plants) vary between 0.01–100 MP m^−3^ [5]. These numbers can certainly be contested owing to the different methodologies applied for sampling and post-collection handling. However, it still gives a perception of the scale; e.g., comparing MP with phytoplankton and zooplankton that have concentrations between 5–100’s µg m^−3^ in different aquatic systems [242], and are also known to bioaccumulate contaminants (both metals and organics). These concentrations of phyto and zooplankton will translate into a much larger surface-to-volume ratio, and the plankton’s ability for active uptake. Considering these facts, should one consider MP as a more important vector for contaminant transport than phyto-zooplankton?

A large number of studies are providing information on the presence of MP in biota with the transfer of MP along the trophic chain, oxidative stress, reduced reproductive capacities, and maternal transfer to name a few. Much of this information is coming from laboratory studies that are working with non-environmental concentrations; in simple terms force-feeding organisms in aquaria with microbeads, fragments and demonstrating the stress. This approach needs serious reconsideration. How likely is it that a fish in open water is going to prey on MP? Based on reported aquatic MP concentrations, the chances are 1 in 10^6^ or less, which raises the question of how legitimate some superfluous statements of reduced biodiversity due to MPs are. Some of the authors have gone to the extent that plastic will outweigh biomass in the ocean by 2050 [243]. These are many journalistic news statements that have created an alarm resulting in substantial funding being diverted to MP studies. Organizations like United Nations Environment Program have launched funding to address the issue. The International Atomic Energy Agency has launched an International Project “NUTEC PLASTIC”. The craze has led resulted in several specialized research groups been established in different countries primarily as a result of the voluminous literature that has been created sometimes by people who do not fully understand the issues.

Many of the studies assessing MP in biota have looked at the digestive system/gut content, and most of them have concluded an effective clearance mechanism [218,223,231,244,245,246,247,248]. Ingested MPs are defecated within one to 72 h raising the question, will this allow any chemical to be leached from the MPs? The findings to date are not sufficient to prove the hypothesis of chemical leaching following ingestion of MPs. Substantial efforts are required to undertake an epidemiological assessment of microplastic effects on human health.

Another important issue that needs some discussion among environmentalists is whether polymeric characterization is a must? Will it have any implication on MP as a vector for contaminants or as a contaminant itself? A huge amount of resources are sometimes spent on polymeric characterization and, as an alternative, we believe a hot needle test should be sufficient to prove the presence of MP.

Some of the published literature has also started mentioning nanoplastic, although it is a very new field of interest and still needs to have consensus on the cutoff size. a widely acceptable size range for NP is between 1 nm and 100 nm [122]. The amount of nanoplastic in the environment is not known, mainly due to technological limitation; There are no marine observational survey techniques that can be used for the monitoring of any nanoparticles in the environment [158]. Some bulk samples can be used to isolate nanoplastics. There are a few techniques such as dynamic light scattering (DLS), nanoparticle tracking analysis (NTA), differential sedimentation centrifugation (DSC), and electron microscopy that are used to quantify the amount and size of nanoparticles in general. However, none of these techniques have been standardized for nanoplastic, with lack of capability to identify polymer. How do we retrieve a nanoplastic particle from an environmental sample? Most of the studies looking at the effects of NPs are laboratory experiments where an extremely unrealistic quantity is added to tissues to see oxidative stress, 5–25% NP weight added of tissue weight % is going to show a sign of stress for sure. What are the environmental concentrations of NPs; this is still to be established. Will researchers also want to know the polymeric composition of NP? If so, how will they do this since micro-Raman and micro-FTIR will not work for this size fraction. In that case, will we have enough material for pyro-GC to characterize polymers?

We believe there is a legitimate need to assess the potential threat from MPs and NPs, however, are we putting too many resources into an environmental concern that has indeed received immense media attention, but still has so many unresolved issues of sampling, identification, characterization and harmonization of protocols associated with it; we have made some suggestions in our previous communications on the need of harmonization of protocols. Some suggestions are made (Supplementary Materials Table S1). This concern can be further appreciated from the results of an interlaboratory comparison (ILC) exercise for MP quantification [249]. A standard sample was provided in form of a 1 L seawater sample with polypropylene and low density polyethylene and high density polyethelene in two plastic bottles. There was a considerable discrepancy in the reported results, laboratories have both over and underestimated. It was observed in this ILC that MPs < 1 mm were underestimated by about 20%.

In another ILC the target was to identify the polymers type, number or mass of MP [250]. In this ILC 12 examples were distributed, six with different types of polymers, five with smaller MPs with soda tablets and a blank soda tablet. The accuracy of polymer characterization in pre-production pallets was between 59–100%, whereas in dissolved soda tablets it was 29–91%. This significant uncertainty further necessitates the need to organize ILCs and look at the produced data with some degree of caution. Another serious consideration is the possible manufacture of standard reference material for MP enumeration and polymeric characterization. Furthermore, we wish to initiate a discussion on the legitimacy of using fresh polymers for undertaking toxicity experiments since most of the secondary MPs are both physically and chemically degraded. Similar consideration should also be given to the manufacture of reference material.

## Data Availability

The data is available in Supplementary file.

## Supplementary Materials

The following supporting information can be downloaded at: https://www.mdpi.com/article/10.3390/ani12030297/s1, Table S1: Summary of the microplastic protocols for different environmental samples.
