# Supplementary material for: Micro-Nano Plastic in the Aquatic Environment: Methodological Problems and Challenges
Source: Animals (Basel). 2022 Jan 25;12(3):297. doi: 10.3390/ani12030297 (PMC8833669; doi:10.3390/ani12030297)
Supplement: Supplementary file 1 [file animals-12-00297-s001.zip › animals-1464987-supplementary.pdf]

Supplementary Table S1. Summary of the microplastic protocols for different environmental samples.

Sample	Sample collection	Sample preparation	Identification	Reporting units
Seawater	45 µm mesh size plankton/neuston net equipped with a flow meter	An initial digestion with 10% KOH solution in a 1:3 ratio (sample: solution ratio) to digest the biological material at 40°C for 72h; followed by a second step if needed to treat the sample with 15% H_2O_2 in 1:1 ratio to oxidize the organic matter at 40°C and staining using Nile Red.	Visual examination: Stereomicroscope, SEM; polymeric characterization using µ-Raman, µ-FTIR, ATR-FTIR, pyro-GC-MS.	MP m^{-3} and g MP m^{-3}
Intertidal sediment	Transect perpendicular to beach from high to low waterline; samples collected using a wooden or metallic frame, top 5 cm should be collected using stainless steel spoon			MP cm^{-3}, MP kg^{-1} dry and MP m^{-2}
Bottom sediment	Collected using a Van Veen grab, barrel type sediment gravity corer, or a box corer. However, we recommend using a barrel type gravity corer of 10 cm in diameter.	The precise weight and moisture content for each core slice should be obtained for sediment cores. In case of using a Van Veen grab, a bulk sample after the discharge of water, should be transferred into labeled glass jars, or an aluminum dish.		
Phyto-plankton	Samples collected with plankton net of 20 µm or 45 µm mesh size, with a net diameter opening of 50-60 cm diameter and 2.0 m in length equipped with a flow meter.	The collected samples are preserved with 4% acidified Lugol's solution or formalin. An initial digestion with 10% KOH solution in a 1:3 ratio (sample: solution ratio) to digest the biological material at 40°C for 72h; followed by a second step if needed to treat the sample with 15% H_2O_2 in 1:1 ratio to oxidize the organic matter at 40°C, and staining using Nile Red.		MP per sample; MPg^{-1} tissue
Zooplankton	Sample collected using 110-µm mesh size plankton net with 0.6 m mouth diameter and 2.0 m in length is recommended, although finer mesh sizes can be used. The samples should be collected by making vertical plankton tows from the deepest portions of the water column to the surface.	Samples are frozen at -20°C until sample preparation. An initial digestion with 10% KOH solution in a 1:3 ratio (sample: solution ratio) to digest the biological material at 40°C for 72h; followed by a second step if needed to treat the sample with 15% H_2O_2 in 1:1 ratio to oxidize the organic matter at 40°C, and staining using Nile Red.		
Fish and shrimp	Samples collected using trawl nets and preserved in liquid nitrogen. However, the size and weight of an individual specimen should be taken into account.	All the samples obtained for single species can be pooled and frozen at -20°C until sample preparation. An initial digestion with 10% KOH solution in a 1:3 ratio (sample: solution ratio) to digest the biological material at 40°C for 72h; followed by a second step if needed to treat the sample with 15% H_2O_2 in 1:1 ratio to oxidize the organic matter at 40°C, and staining using Nile Red.		
Bivalves & gastropods	Bivalves can be hand collected. Other benthic organisms such as marine worms can be hand collected by divers or by sediment grab sampling.			
Aerosol	Depending on the aim of the study, either passive or active collection techniques are used. We recommend deploying both active and passive samplers concurrently. Active sampling should be carried out using a low- or high-volume air sampler with at least a six-stage cascade impactor. We recommend using inorganic filters (quartz fiber, alumina or silver).	The aerosol samples include inorganic and organic matter. We recommend the use of 5 – 15% H_2O_2. Following digestion of organic matter, density separation using NaCl and KCl solution and filtering onto 0.45 µm pore size filters. Samples are then transferred to silver membrane filters (1.2 µm pore size), placed in glass beakers with 10 mL 10% HPLC-grade methanol in ultrapure water and extracted by standing beakers in a sonicating bath for 1 min. The sample is then stained using Nile Red.		MP m^{-3}, MP m^{-2}. $Weight_{MP}$ $mg^{-1}_{aerosol}$
